# An Obsessive-Compulsive Symptom-Related Headache in a Patient with Schizophrenia

**DOI:** 10.1155/2020/8824204

**Published:** 2020-10-10

**Authors:** Hsinsung Yuan, Junjun Liu

**Affiliations:** Nanjing Meishan Hospital, Nanjing, China

## Abstract

Obsessive-compulsive symptoms are prevalent, manifold, and sometimes insidious in patients with schizophrenia. In this case study, we reported an intractable headache that bears a close relationship with obsessive-compulsive symptoms in a schizophrenia patient. In a series of treatments, the headache was miraculously susceptible to haloperidol treatment.

## 1. Case

A 52-year-old woman with a 9-year history of schizophrenia presented with a persistent headache and depression after she was mauled by five people 2 months ago, with the left side of the top of her head hit by a crabstick and no disturbance of consciousness after the attack. She had two head MRI scans after the hit, but all of them showed negative results. The woman presented argumentative hallucinations and paranoid delusions 9 years ago. She was diagnosed with schizophrenia and was then treated with 600 mg quetiapine daily. The treatment was effective, and quetiapine was maintained at a dosage of 200 mg daily. During these 9 years, she sometimes heard some people talking about her, but even if she heard this, she thought these voices had nothing to do with her. She worked as a cleaner during these years. She has been hard-working and self-disciplined. The hallucinations increased in both frequency and intensity 6 months ago, and she could constantly hear her neighbours and people from nearby villages commenting on her. Two months ago, a white dog and a yellow dog were fighting in front of her house, and then her neighbour shouted at these dogs. At that time, the patient was dressed in yellow, and she thought her neighbour was shouting at her, so she went to fight her neighbour; then, the neighbour's family came out and mauled her. As an outpatient, she had taken 15 mg olanzapine daily, 10 mg aripiprazole daily, and 100 mg sertraline daily during the following two months, but her headache had worsened.

When the patient came to the inpatient ward, she was kempt but made passive contact, talked little, and avoided eye contact with psychiatrists; her only complaint was a severe and unbearable headache. Her thoughts were consistent and logical, but her reaction was slow. She could hear several different kinds of auditory hallucinations. The first was a kind of functional auditory hallucination triggered by whispering, barking, or car noises, and these whispers could present as argumentative swearing. The second was a kind of primary auditory hallucination, which could occur without any sign, and it could be combined with a persecutory delusion in which she thought there were people nearby talking about her. The third kind of functional hallucination was that when she talked with psychiatrists, she could hear psychiatrists swearing at her at the same time as the real psychiatrists' voice. The fourth kind of hallucination was a psycho-hallucination that stemmed from her brain. These voices often commented on her or presented themselves as the fifth form and broadcasted her thoughts, which coincided with the dilution of the diffusion of her thoughts. When she heard the commenting and criticizing voice, thoughts of guilt came to her mind. When thoughts of guilt emerged, she denied them and swore against them in her mind, but she could not avoid these thoughts. Every time she swore against these people, she felt shameful and repentant because she believed that speaking ill of others was immoral; she then was angry with herself, and her headache then appeared. When she lived in the hospital, she often wandered around the garden, which she said could relieve her headache because she could verify that nobody was talking about her in the garden. She had insomnia because the auditory hallucinations were too loud to allow her to sleep, and in the early morning, the auditory hallucinations interrupted her sleep.

She did not smoke, did not drink alcohol, and denied the use of illicit drugs. No parental relative was reported to have a diagnosis of mental disorder. There was no comorbid physical illness. The patient only completed 3 years of elementary education. She quit her studies because her family could not afford her tuition fee, and she was required to work to support her family; however, she could read and write. She had been obedient and forbearing since she was a child. She was married at 24 years of age in an arranged marriage. Her first child was a daughter, so she gave birth to a son one year later because of patriarchal culture. However, owing to the one-child policy, she had to pay a large amount of forfeit, and it took her 10 years to repay the debt. Unfortunately, her son accidentally drowned at 7 years old. After her son died, she had an episode of depression. At that time, she had feelings of sorrow and self-condemnation. She laid on the bed every day and quit her job because she heard many colleagues talking about her. She was diagnosed with major depressive disorder, but she did not take medicine, and her depression subsided 6 months later. During these 14 years, she had not shown any sign of mood or psychotic symptoms, and her symptoms during these 9 years was not closely related to the context of her son. She did not avoid to talk about her son, and her son's death no longer bothers her. There was no obvious manic episode in her life.

On examination, her head MRI scan was normal. Her blood count, liver function, kidney function, thyroid function, and infectious disease portfolio were normal. Her Hamilton Anxiety Scale (HAMA) was 23, and her 17 items of Hamilton Depression Rating Scale (HAMD-17) was 24. We conducted drug-gene testing, which showed that her CYP2D was ∗10/∗41 and her FKBP genes were GG (rs4713916) and CC (rs1360780), which implied that venlafaxine might not be effective.

## 2. Discussion

Schizophrenia is regarded as a disorder with hypoalgesia [1–4]. However, headache has been regarded as a widely reported symptom of schizophrenia [5–9]. Studies have shown that 57% of schizophrenia patients suffer from headache, but most of these patients cannot be classified as having any kind of headache in the International Classification of Headache Disorders (ICHD); furthermore, these headaches are not related to any social, demographic, and biological factors, including whether participants adhere to their treatment [10]. Furthermore, it remains controversial whether headaches are a manifestation of mental symptoms or a sign of physical injury [11]. After she was hit two months ago, she presented a variety of symptoms amongst headache and depression. The depressive episode during these two months might be related to the acute stress condition. However, she was not physically injured by that incident, so it was possibly that acute stress condition was owing to her delusion. In this case, we present a headache that is closely related to obsessive-compulsory symptoms in schizophrenia.

In patients with psychotic symptoms, depression, obsessive-compulsory symptoms, and headache, we have to differentiate schizoaffective disorder and bipolar disorder. In this case, though the woman's medication had been maintaining at a low dosage and she had presented depression and headache in this episode; she had been having residue persistent paranoid auditory hallucination during the last nine years, with a flat mood without comorbid personality disorder. Even in this episode, her hallucination has already been worsening 6 months ago and she presented a formal thought disorder in that she considered her neighbour was shouting at her. According to ICD-10, the patient's psychotic symptoms have been persistent and worsened 6 months ago, but the depression has only presented since 2 months ago; the patient did not meet the criteria of schizoaffective disorder. However, according to ICD-11, the diagnoses of schizophrenia and schizoaffective disorder are intended to apply to the current period of illness, so she could meet the criteria of schizoaffective disorder under the ICD-11 criteria.

Some case studies have reported that mood stabilizers [12] and olanzapine [13] were associated with alleviating headache in schizophrenia. We used 1000 mg sodium valproate daily to decrease her agitation with regard to her headache, which often occurred when she was agitated, and it was also reported that this drug is helpful for both headache and functional hallucination in schizophrenia. After using this drug, the patients' headache and agitation were slightly alleviated, but the headache still persisted after one week of medication, with no improvement in obsessive self-accusatory thoughts.

Obsessive-compulsive symptoms (OCS) in schizophrenia are common but challenging to manage. Twenty-five percent of schizophrenia patients present OCS [14–18], and it is arguable that clozapine, one of the most effective medications in schizophrenia, could worsen the symptoms [19]. In contrast, it has been reported that OCS in schizophrenia could be relieved faster than OCS in OCD [20]. In this patient, the usage of olanzapine slightly alleviated auditory hallucinations, but after the hallucinations ceased, the headache remained and even worsened. We found that the headache was closely connected with a series of obsessive self-accusatory thoughts and agitated reactions, which could be obfuscated with depression and potentially lead psychiatrists to add clozapine.

We found that the patient's silence and avoidance were evidenced not through her negative symptoms and depression but rather through her endurance of her anger and agitation. In regard to her working performance before hospitalization, her negative symptoms did not appear to progress, so we tapered off the ineffective aripiprazole treatment. Although olanzapine could alleviate OCS in schizophrenia [21], to alleviate her obsessive thoughts, we started the treatment of haloperidol; haloperidol and amisulpride are the two main drugs used to control OCS in schizophrenia [22]. With regard to the genetic results, venlafaxine was not suitable for this patient, and we used escitalopram to address the OCS and sad mood [23]. One week after the usage of 2 mg haloperidol and 10 mg escitalopram daily, all auditory hallucinations and delusions disappeared, and the obsessive self-accusatory thoughts and headaches disappeared.

Compared with depression in affective disorder, depression in schizophrenia is characterized by self-accusatory thoughts, thoughts of guilt, hypochondriasis, and delusions, rather than hypodynamics in MDD [24]. In the case of this patient, she denied feeling lethargic, had obvious agitation, and worked very hard during the peak of her depression. It must be stated that her self-accusation is a form of obsessive-compulsive symptoms, but regarding personality traits, she avoided people, talked less to hide her agitation, and probably even developed a reaction mechanism, namely, somatization, which is a psychoanalytic term; somatization often develops in Chinese women in rural areas. If her depression and negative symptoms were recognized, the use of aripiprazole and a relatively large dosage of an antidepressant, or even clozapine, would worsen her compulsive symptoms and agitation.

## 3. Patient Outcome

After the addition of 1000 mg sodium valproate daily and the reduction of her dosage of antidepressants to alleviate her agitation, her headache was slightly alleviated, and she reported that her feelings of anger and agitation were significantly reduced. However, after one month, she still had many auditory hallucinations, as well as obsessive thoughts of self-accusation, and she still compulsively swore at them. She reported that when she saw other people, including psychiatrists, their images would reflect in her brain, and she could not dismiss these images. Then, we changed aripiprazole to 2 mg haloperidol per day and stopped venlafaxine to alleviate OCS. All her psychotic and obsessive-compulsive symptoms rapidly resolved over the next week; she said she no longer heard any auditory hallucinations arguing about or commenting on her. She denied being able to see another person's image, her thoughts were no longer broadcasting, and her depressive mood and headache disappeared. At the same time, her HAMA score was 2, her HAMD-17 score was 2, and she was discharged from our hospital. One month after her discharge, the patient relapsed due to a mild obsessive thought and secondary depression, in which she felt her boss was not satisfied with her work; she then felt remorseful, so we increased her dosage of haloperidol to 4 mg per day. Three months, six months, and one year later, most of the patient's symptoms were in remission, with some auditory hallucination persisting, but she could address them. She has kept taking 1000 mg sodium valproate daily, 4 mg haloperidol per daily, and 15 mg olanzapine daily. She maintains the ability to work and maintains an acceptable quality of life.
